# Magnetoimpedance Thin Film Sensor for Detecting of Stray Fields of Magnetic Particles in Blood Vessel

**DOI:** 10.3390/s21113621

**Published:** 2021-05-22

**Authors:** Grigory Yu. Melnikov, Vladimir N. Lepalovskij, Andrey V. Svalov, Alexander P. Safronov, Galina V. Kurlyandskaya

**Affiliations:** 1 Institute of Natural Sciences and Mathematics, Ural Federal University, Ekaterinburg 620002, Russia; grigory.melnikov@urfu.ru (G.Y.M.); vladimir.lepalovsky@urfu.ru (V.N.L.); safronov@iep.uran.ru (A.V.S.); Andrey.svalov@urfu.ru (A.P.S.); 2 Departamento de Electricidad y Electrónica, Universidad del País Vasco UPV/EHU, 48080 Bilbao, Spain

**Keywords:** magnetic nanomaterials, magnetic multilayers, magnetic particles, magnetic composites, magnetoimpedance, stray fields, biomedical applications, COMSOL modeling

## Abstract

Multilayered [FeNi (100 nm)/Cu (3 nm)]_5_/Cu (500 nm)/[Cu (3 nm)/[FeNi (100 nm)]_5_ structures were used as sensitive elements of the magnetoimpedance (MI) sensor prototype for model experiments of the detection of magnetic particles in blood vessel. Non-ferromagnetic cylindrical polymer rod with a small magnetic inclusion was used as a sample mimicking thrombus in a blood vessel. The polymer rod was made of epoxy resin with an inclusion of an epoxy composite containing 30% weight fraction of commercial magnetite microparticles. The position of the magnetic inclusion mimicking thrombus in the blood vessel was detected by the measurements of the stray magnetic fields of microparticles using MI element. Changes of the MI ratio in the presence of composite can be characterized by the shift and the decrease of the maximum value of the MI. We were able to detect the position of the magnetic composite sample mimicking thrombus in blood vessels. Comsol modeling was successfully used for the analysis of the obtained experimental results and the understanding of the origin the MI sensitivity in proposed configuration. We describe possible applications of studied configuration of MI detection for biomedical applications in the field of thrombus state evaluation and therapy.

## 1. Introduction

Nanostructured magnetic materials have become a subject of interest in the last decades. In many senses, their popularity is based on the development of new fabrication techniques and significant improvements of existing synthesis methods [1,2,3]. They attract special attention from both the point of view fundamental physical and chemical properties and variety of practical applications [3,4,5]. Among other systems magnetic nanoparticles were in focus for their investigation in technical devices [6,7,8] and medical conditions [9,10]. Medical applications require the most strict protocol for nanomaterials and nanodrugs processing [10]. The key point is evaluation of the same parameter by different techniques and thorough characterization of nanodrugs at all stages of production, transportation and diagnostics or therapy. A very special need in fabrication techniques with well controlled conditions of elaboration of large batches become very clear [10,11]. The majority of the methods of fabrication of magnetic nanoparticles (MNPs) are chemical techniques [12]. However, the electrophysical techniques of the electric explosion of wire or laser target evaporation provide the largest single batches and green synthesis processing with low amount of required solvents [11].

The second important type of magnetic nanostructures is thin films and multilayered structures. They are most often used as sensitive parts of magnetic field sensors and biosensors [13,14]. One of the reasons of special success of magnetic films for sensor applications is their compatibility with existing semiconductor electronics and high potential of packaging [15]. There are different magnetic effects observed in thin film structures capable of supporting the development of magnetic field sensors with sensitivity sufficient to detect either biomagnetic fields or stray fields of magnetic nanoparticles used as biomolecular labels [16]. Their efficiency and choice depend on particular conditions of the detection and many parameters can contribute to the decision and device design. It might be a corrosion stability in biological fluids, power consumption, optimum response in the temperature range under consideration and many others. Importantly, in the case of small magnetic field detectors the sensitivity with respect to applied magnetic field in the field range of a few oersted is a key parameter [17,18]. Among others, magnetoimpedance effect (MI) observed in different magnetic materials [19] was shown to be effective for magnetic biosensing both biomagnetic fields and stray fields of magnetic particles [20].

One of important features of present day nanomaterials applications is their operation in conditions when at least two magnetic components are involved in the technological or biomedical process. The search for optimum conditions of the application (optimum functional properties) becomes quite complex because the best conditions for one of the materials might be far from being optimum for another one. It this case the whole system optimization goes in a direction of the search for most favorable work interval in which each one of the materials might be functioning not with the best but acceptable performance. The simplest example of such system is a magnetic particle creating stray fields in the external magnetic field and magnetic field sensor. Application of external magnetic field increases the magnetic moment and the value of the stray fields created in the presence of magnetic particles: there is an extended interval in which the rule “the higher the external field the higher the stray fields” is correct. However, external magnetic field detector may have the highest sensitivity with respect to the applied magnetic field in the field range where the stray fields of the particle are still too small to be detected. In this case magnetic label detection become impossible. Summarizing, in each particular application involving two or more magnetic materials there is a goal to search for synergetic combinations in order to reach the best performance.

Nowadays magnetic field sensors attract attention for a variety of applications related to detection of biocomposites containing magnetic nano- or microparticles. The possibility of the detection of superparamagnetic nanoparticles of iron oxide by MI multilayered sensitive element was shown in the case of such biomimetic materials as ferrogels [18,21]. Those measurements were made in close proximity mode: the ferrogel sample was placed directly onto the surface of MI multilayered sensitive element. However, the sensitivity of MI detectors should be sufficient for magnetic particles (MPs) detection at the distances of the order of 100 microns including detection of the presence of aggregates of a complex geometry.

One of such applications is a detection of MPs agglomerates in the blood vessel. A pathological blood clot that forms during the life of a patient in the lumen arteries or veins is denominated as a thrombus. Diseases caused by thrombosis are from the list of the leading causes of death. Thrombosis is currently being treated by surgery, which consists in performing a complex operation with a high-risk complication. Another way is the therapy with the use of thrombolytics medication, having many side effects. One of the ways to reduce side effects is targeted drug delivery to a blood clot using MPs [22]. For example, biocompatible MPs of magnetite can be employed [2,21]. During the therapy it is critically important to determine the local concentration of magnetic particles in the thrombus region. Here we propose the next step of the development of this interesting idea. The task of the local concentration definition can be solved using a MI sensor, detecting stray fields of magnetic particles forming agglomerates in the thrombus area.

The aim of this work is to study the effect of stray magnetic fields of microparticles of iron oxide in a model geometry imitating a blood clot in a blood vessel. It is done by experimental study of magnetoimpedance responses of multilayered sensitive elements based on permalloy at different positions magnetic sample made of composite material (epoxy resin and magnetite particles). In addition, Comsol modeling of stray fields is used for the analysis of the obtained experimental results.

## 2. Materials and Methods

### 2.1. Materials

**Figure 1:**
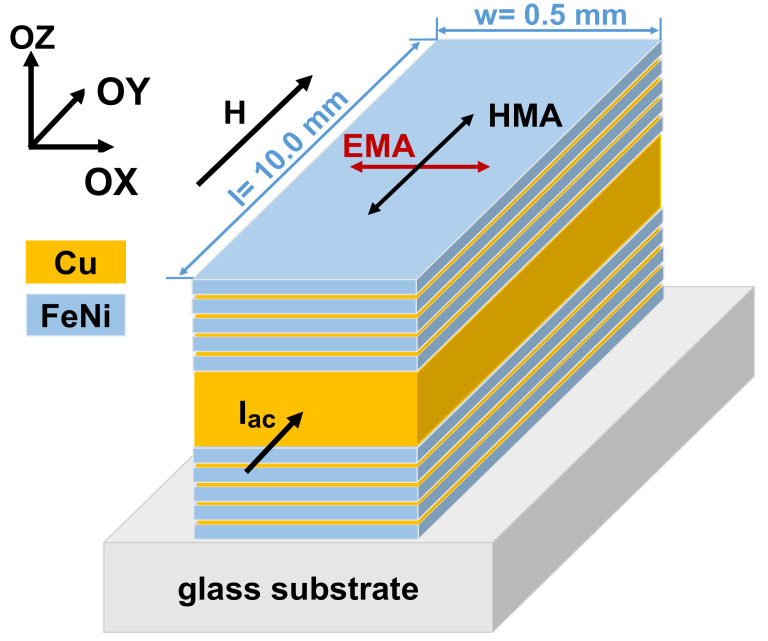
Scheme of multilayered [FeNi (100 nm)/Cu (3 nm)]_5_/Cu (500 nm)/[Cu (3 nm)/[FeNi (100 nm)]_5_ MI sensitive element. EMA—easy magnetization axis; HMA—hard magnetization axis; I_ac_—high frequency alternating current flowing along the rectangular MI element; H—external magnetic field applied during magnetic measurements. Technological magnetic field during deposition was applied along the short side of the MI element.

For magnetoimpedance sensitive element, we selected configuration with open magnetic flux (Figure 1) [21]. It consisted of a number of layers of the same width and length deposited onto the glass substrate one on the top of the other (Figure 1). Metallic masks were used during the magnetron sputtering deposition of the layers. The MI element was deposited through the mask with the shape of the stripe (0.5 × 10 mm) by dc magnetron sputtering onto a glass substrate. The deposition was prepared with a background pressure of 3 × 10^−7^ mbar and a working Ar pressure of 3.8 × 10^−3^ mbar. During the deposition process an in-plane constant magnetic field H_d_ = 100 Oe was applied along the short side of the MI elements for inducing a transverse uniaxial in-plane magnetic anisotropy. Therefore, the direction parallel to the short side of the rectangular stripe along which the technological magnetic field was oriented during film deposition was expected to be an easy magnetization axis (EMA). The direction along the long side of the rectangular MI element was expected to be the hard magnetization axis (HMA). The deposition rates for each part of the multilayered structure (copper and permalloy) were calculated in previous calibrations. As it was proposed in previous works [21,23,24,25] the magnetic layers of permalloy before and after the Cu-lead were nanostructured by Cu-spacers in order to avoid the transition into a “transcritical” state [26,27,28].

Figure 1 shows the general arrangement of the layers of magnetoimpedance [Fe_21_Ni_79_ (100 nm)/Cu (3 nm)]_5_/Cu (500 nm)/[Cu (3 nm)/[Fe_21_Ni_79_ (100 nm)]_5_ elements with a copper conductive central lead. One can see that the used configuration is a symmetric MI structure.

In addition, despite the complexity of the structure it consists of the magnetic layers of two compositions: Cu and FeNi. For their deposition pure Cu and alloyed FeNi targets were used. Although in our previous works discussing both experimental and theoretical results for FeNi/Ti and FeNi/Cu based systems the higher MI variations were found for FeNi/Ti based multilayered element [21,29], FeNi/Cu based elements are much easier to fabricate.

Cylindrical polymer rod with the diameter of about 5.1 mm and approximately 54 mm length was used for model experiments. The sample mimicking thrombus in a blood vessel was made of epoxy polymer, and it was combined of three sections along its length. Both end sections (25 mm each) were non-ferromagnetic epoxy polymer, the central section (4 mm) was a ferromagnetic inclusion. It was made of a filled epoxy composite containing 30% weight fraction of commercial (Alfa Aesar, Ward Hill, MA, USA) magnetite submicron sized particles (Fe_3_O_4_ phase—94 weight%; Fe_2_O_3_ phase—1 weight%; FeO(OH) phase—5 weight%). According to SEM microphotographs the particles were quasi-spherical and their caliper diameter fall within 0.05–0.50 micron with a median value 0.30 μm. Saturation magnetization of magnetite particles was 84 emu/g, remnant magnetization was 6.6 emu/g, and coercivity was 78 Oe.

The model sample was prepared with the epoxy resin KDA (Chimex Ltd., St. Petersburg, RF) as a polymer matrix. Epoxy resin was mixed with a tri(ethyl)-tetra(amine) hardener (Epital, Moscow, Russia) in a 6:1 weight ratio. Magnetite particles were vigorously mixed with liquid epoxy resin at 25 °C for 10 min in order to obtain a homogeneous mixture. The curing of the combined model sample was performed in a polyethylene tube in a stepwise manner. At the first step the blank epoxy composition was cured in the tube, which was vertically positioned, then the filled epoxy composition was cured on top of the blank epoxy polymer, and finally the blank epoxy composition was placed and cured on top of the magnetic central section. Each curing step was done for 2 h at 70 °C. The control sample (control) was also prepared by curing the blank epoxy resin cylinder without magnetic particles (0.0% concentration composite).

### 2.2. Experimental Methods

The average grain size of the microparticles and their shapes were evaluated by scanning electron microscopy, SEM (JEOL JSM-7000 F, Japan) for the set of the particles spread onto carbon conductive tape. The magnetic properties of the multilayered sensitive elements were studied using magneto-optical Kerr-microscopy (MOKE). Both magnetic domains and magnetic hysteresis loops were studied using Kerr-microscope (Evico Magnetics GmbH, Dresden, Germany). The magnetic hysteresis loops of epoxy resin and magnetic filled composite were measured at room temperature by a vibration sample magnetometer 7407 VSM (Lake Shore Cryotronics, Westerville, OH, USA).

**Figure 2:**
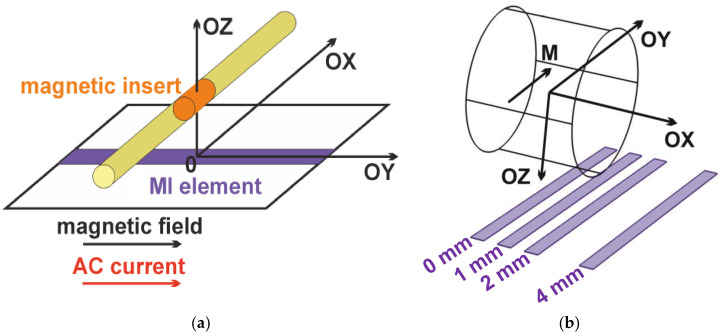
Scheme of the model experiment for detection of magnetic insert mimicking thrombus in the blood vessel by a magnetoimpedance multilayered sensitive element (**a**). Scheme of Comsol modeling of the stray fields of the cylindrical sample of magnetic composite. Positions at the distances of 0, 1, 2, and 4 mm along the OX direction are shown as different rectangular elements for obtaining a visual effect. Magnetization value M = 2.5 G (**b**).

The longitudinal MI effect was measured, i.e., external applied magnetic field (H) and the high frequency alternating flowing current (AC) were parallel to each other (Figure 2). MI measurements were made by the automatic system based on Agilent HP E 4991 An impedance analyzer (Agilent, Santa Clara, CA, USA) at room temperature in an external magnetic field created by Helmholtz coils. The in-plane magnetic field in the range of ±150 Oe was applied along the long side of the MI element in the direction of the alternating current flow for the currents in the frequency range of 1–400 MHz. This frequency range was selected taking into account both the length of the MI element and its expected sensitivity with respect to an applied field [21]. As before, the system was carefully calibrated for the extraction of the intrinsic impedance values [15,21].

The experimental detection of the magnetic insert mimicking thrombus in blood vessels was carried out as a model experiment as follows. The MI element was incorporated into a “microstripe” line using silver paint. The impedance of the MI element was measured at various positions of the center of the magnetic inset with respect to a center of the MI element. The magnetic insert inside the non-ferromagnetic tube was placed at a distance of 1.1 ± 0.2 (mm) above the MI element surface (Figure 2). The tube was parallel to the surface of the MI element and its axis was perpendicular to the long side of the element. The tube and the magnetic insert can be displaced along the axis perpendicular to the long side of the MI element (along the OX axis).

Magnetic composite inset was previously magnetized in a high external magnetic field up to 10 kOe, resulting in the remnant magnetization of the sample due to the magnetic hysteresis [30]. During the experiment, the remnant magnetization (*Mr* = 2.5 G) of the inset was directed along the OY axis and remained constant in the external field range. The zero position (an origin) was considered to be the center of the MI element (Figure 2).

For the MI response description, we used the magnetoimpedance ratio (MI ratio) (1), the MI ratio sensitivity (2) and the MI response (3) which were calculated as follows:

(1) $$\Delta Z/Z=100\times \frac{Z\left(H\right)-Z\left(H_{max}\right)}{Z\left(H_{max}\right)}$$

(2) $$S\left(\Delta Z/Z\right)=\frac{\delta \left(\Delta Z/Z\right)}{\delta H}$$

(3) $$\Delta \left(\Delta Z/Z\right)=\Delta Z/Z_{control}-\Delta Z/Z_{position}$$

where $H_{max}$ = 100 Oe and $\delta \left(\Delta Z/Z\right)$ is the change MI ratio for the total impedance at the change of the magnetic field δH = 0.1 Oe,$\;\Delta Z/Z_{control}$ is the total impedance MI ratio with the control;$\;\Delta Z/Z_{position}$ is the total impedance MI ratio with the magnetic insert located in the certain position. For MI frequency dependence analysis, the change of the maximum value of the total impedance $\Delta Z/Z_{max}$ was provided. The experimental error in the impedance determination was within 1%. To estimate the random error in the setting of the magnetic insert position, the experiment was carried out three times on different days. Random error was estimated as follows:

(4) $$\dot{\Delta }\left(\Delta Z/Z\right)=\sigma \times t$$

where σ—standard deviation of ΔZ/Z, t = 4.3—Student coefficient of t-distribution for n = 3 [31].

### 2.3. Magnetic Stray Field Distribution Modeling

Nowadays computer modeling becomes more and more requested in the cases when magnetic filled composites are involved in the detection process. Among other reasons of usefulness of them we would like to mention the fact that the existing techniques of the structural evaluation of magnetic materials based on magnetic nanoparticles in many senses are still under development [32,33]. The magnetic stray field distribution of the magnetic insert on the surface of the MI multilayered sensitive element was modeled in Comsol MultiPhysics (AC/DC Module) (Comsol LLC, Switzerland, License № 17074991). In the Comsol model, *y*—the component of the magnetic field *Hy* along the long side of the MI element and *x*—the component of the magnetic field *Hx* along the short side of the MI element in XY plane were calculated. The geometries of MI element (0.5 mm × 10.0 mm) and the magnetic insert diameter 5.0 mm were taken to be the same as in the experimental MI measurements. The origin was set in a center of the magnetic insert and coordinates of the MI element center defined the position of the MI element. The magnetic distribution was calculated at different MI element positions along the OX axis (0.0, 1.0, 2.0 and 4.0 mm—these positions are shown as different rectangular elements for obtaining a visual effect), the OY coordinate 0.0 mm, the OZ coordinate 1.1 mm (Figure 2b).

The magnetization of the magnetic insert (M) was directed along the OY axis and according to the experimental data for hysteresis loop measurements M(H) it was about 2.5 G in the zero field (remnant magnetization). The detailed discussion of the magnetic properties of the filled composite sample will be given in Section 3.

## 3. Results

### 3.1. Structure and Magnetic Properties

**Figure 3:**
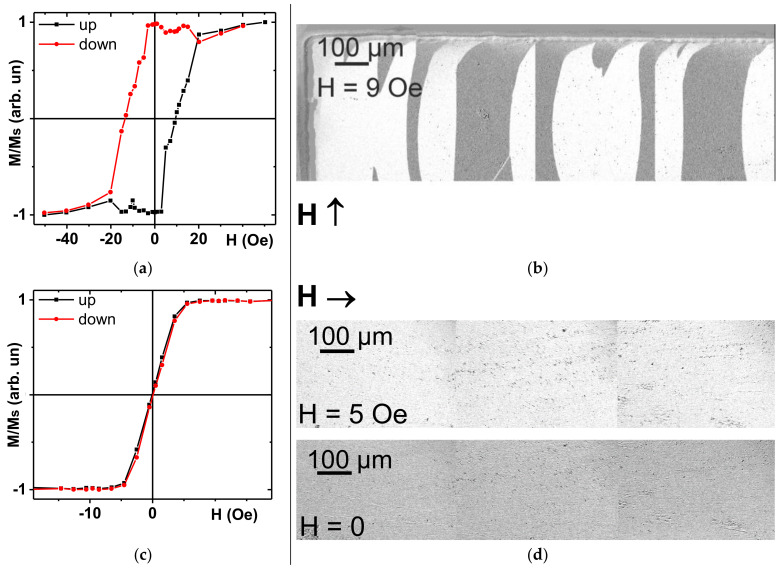
Magnetic hysteresis loops and selected examples of magnetic domain structures of MI element measured by Kerr–microscopy. (**a**,**b**) Along the short side of the MI element (EMA) or (**c**,**d**) long side of MI element (EMA). Surface magnetic domain images for the MI element: magnetic field of 9 Oe (coercive force) applied along the EMA (**b**); magnetic field of 5 Oe (approaching the anisotropy field) or close to zero applied along the HMA.

**Figure 4:**
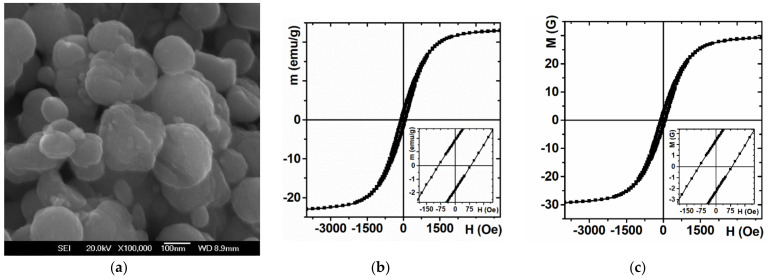
General view of magnetic particles used as a filler for fabrication of the epoxy composites—SEM (**a**). Magnetic properties of the magnetic composite: (**b**) specific magnetic moment; (**c**) magnetization.

Figure 3 shows the hysteresis loops and selected examples of magnetic domain structure of multilayered [FeNi (100 nm)/Cu (3 nm)]_5_/Cu (500 nm)/[Cu (3 nm)/[FeNi (100 nm)]_5_ MI sensitive element measured along EMA and HMA. It is clearly seen that deposition in an external magnetic field resulted in formation of well-defined uniaxial induced magnetic anisotropy along the short side of the rectangular stripe. The shapes of the hysteresis loops are in accordance with magnetic domain structures and special features of magnetization processes for magnetization along EMA and HMA. Magnetic domains in zero applied field (after saturation) are typical for soft ferromagnet bar domains with in-plain orientation of magnetization corresponding to opposite directions in “white” and “black” patterns. Closure domains near the borders of the MI element were shown to have complex structure [34] but this point is not under consideration in the present work. Magnetization process along the short side was characterized by domain wall displacements, coercive force defined for the top layer was of the order of 9 Oe (Figure 4b).

It is worth mentioning that magnetooptical signal passes to the permalloy for at most 40 nm. Taking into account direct and return pass, we obtained 20 nm information length [30]. For HMA the magnetization process occurs by the pure rotation for a magnetic field applied along the long side of the MI element. Thus, multilayered nanostructures have induced in-plane uniaxial magnetic anisotropy along the short side of the element and the anisotropy field of about 5 Oe. This ensures the work interval of the magnetic field sensor in the range below 5 Oe being quite convenient for applications.

Figure 4a shows SEM images of iron oxide nanoparticles that were used for fabrication of epoxy composites. Despite the fact that the shape of the microparticles varies, in a most general description we can say that they tend to be quasi-spherical and their average caliper size is about 300 nm. As a result of the analysis of the magnetic hysteresis loops the magnetic properties of the epoxy composite can be described as follows: coercivity *Hc* = 85 Oe, specific saturation magnetic moment *m_s_* = 23 emu/g, residual specific magnetic moment *mr* = 1.8 emu/g (Figure 3a), saturation magnetization *Ms* = 29 G, remnant magnetization *Mr* = 2.5 G (Figure 4b,c).

The specific magnetic moment of the control composite (without magnetic particles) can be described as weak diamagnetic response, which was four orders of magnitude smaller than a magnetic response of the magnetic composite itself. The specific magnetic moment of saturation of magnetic microparticles, calculated from magnetic measurements of the composite and in accordance with the direct measurements of the NPs is of the order of 65 emu/g, which is in accordance with the values for magnetic particles of magnetite of such a size [35].

### 3.2. Detection by MI Sensitive Element

**Figure 5:**
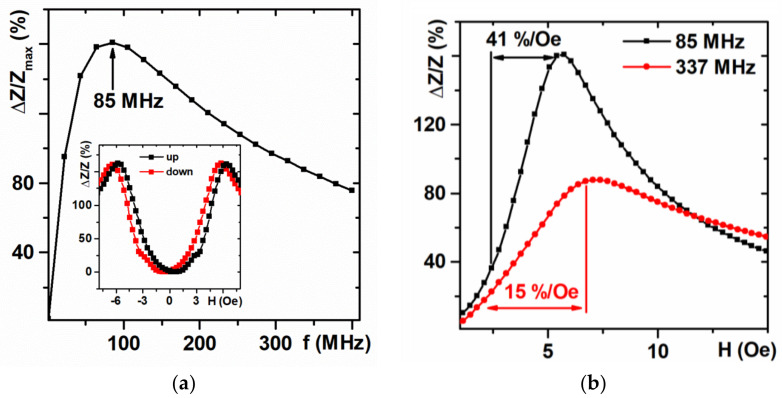
(**a**) Frequency dependence of the maximum value of the MI ratio. In the insert the field dependence of the MI ratio at 85 MHz high-frequency current. (**b**) “Down” curves of field dependence of the MI ratio at 85 and 337 MHz of the driving current frequency.

Figure 5 shows MI responses (maximum MI ratio) of multilayered sensitive element as a function of the frequency of the driving current. For the whole magnetization cycle of the MI element there are “up” (from −150 Oe to 150 Oe) and “down” (from 150 Oe to −150 Oe) branches of the MI curves. Inset for Figure 5a shows the field dependences of the MI ratios for the total impedance. One can see that the field dependence of the MI ratio “up” and “down” curves are “mirror-symmetric” with respect to each other. However, each one of them is not symmetric with respect to the H = 0 axis. Such a behavior was previously observed many times and for each particular MI material and sensitive element the reason or a number of reasons causing such a behavior can be different. We will briefly mention some of the reasons—high order type of effective magnetic anisotropy, longitudinal static magnetic hysteresis, insufficient value of the applied field, etc. [36,37,38] but their discussion is also not considered for the present work.

The maximum MI ratio (close to 160%) observed at the frequency of 85 MHz also corresponds to a maximum of the magnetic field sensitivity (41%/Oe). We define the working interval of magnetic sensor as the field range corresponding to maximum sensitivity: here it is 3–5 Oe. After 87 MHz as the driving current frequency increases, the MI ratio decreases, and the sensitivity and the maximum value of the MI ratio also are decreasing (Figure 5b). Magnetoimpedance is a spectroscopic technique—the measurements can be done and evaluated for a number of the frequencies of the interest. We therefore selected two values of the driving current frequency for the analysis: the frequency for the highest sensitivity with respect to the applied field and high frequency for which dielectric contributions are high but the magnetic field sensitivity of the multilayered element is still sufficient for the detection process.

Earlier we have investigated the MI responses of the multilayered film sensitive elements in the presence of micro- and nanoparticles on their surface or using polyacrylamide gel coverings [15,18]. Gels and ferrogels contain a large amount of water in their structures, contributing to MI signals due to high dielectric constant, which is especially strong at high frequencies. This circumstance defines strict conditions for the quality of the gel or ferrogel samples: their shape and weight must be controlled thoroughly. We therefore decided at the first stage of the development of the model experiments for thrombus detection to use composites with low dielectric response of the matrix. Epoxy resin dielectric constant (ε = 3.49 at 25 °C) is low in comparison with dielectric constant of liquid water being around 78.4 for low frequency ranges below 100 kHz. In a MHz range such a difference becomes even higher. With this selection of the matrix MI measurements become less demanding to the deviation of the weight and the shape of the samples of the composites, the dielectric effect can be neglected as making very small contribution.

**Figure 6:**
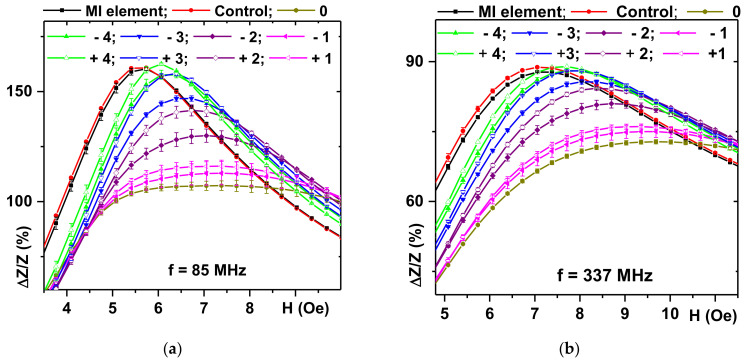
Field dependences of the MI ratio at the driving current frequencies of 85 (**a**) and 337 MHz (**b**) for different positions of epoxy composite samples (see also Figure 2a). MI responses of multilayered [FeNi (100 nm)/Cu (3 nm)]_5_/Cu (500 nm)/[Cu (3 nm)/[FeNi (100 nm)]_5_ MI sensitive element itself and epoxy resin sample without magnetic particles are also given. Numbers indicate displacements in mm of the center of the cylindrical composite from zero position corresponding to the point just above the center of the MI element being elevated by 1.2 mm from the free side of the element.

Further, for simplicity we consider the “down” MI ratio curves only. As the magnetic field composite sample approaches the MI element, the stray magnetic fields of the magnetic particles affecting the surface of the MI element increase. It leads to the MI ratio decreases and MI curves peaks become shifted along the H axis (Figure 6). According to the existing theoretical approaches [21,37,38] such a behavior in the longitudinal MI geometry can be explained as follows. The shift of the MI ratio curves is due to the appearance of the parallel component of the effective magnetic field directed along the long side of the MI element. The decrease in the maximum MI ratio value is due to the perpendicular component of the magnetic field directed perpendicular to the long side of the MI element. The shift of the MI curves without the strong change of the maximum MI ratio (−4 and 4 mm) is similar to the case of an additional shift field opposite to the external magnetic field along the long side of the element. The largest decrease in the maximum value of the MI ratio corresponds to the position when the center of the magnetic filled composite sample is situated just above the center of the MI element.

Maximum variations of the MI ratio in course of the change of the position of the filled composite were observed at the driving current frequency of 85 MHz corresponding to the maximum MI sensitivity with respect to external magnetic field (Figure 6a). In some cases, MI curves related to the symmetric positions of the magnetic composite sample (for example, −3 and 3 mm) do not match to each other. One of the reasons is possible magnetic heterogeneity of the composite as for the filling rather large particles were used. Another reason could be an experimental error in the displacement definition. Despite this uncertainty, the results are very clear. The position of the magnetic filled composite sample was well detected based on the analysis of the maximum value of the MI ratio.

**Figure 7:**
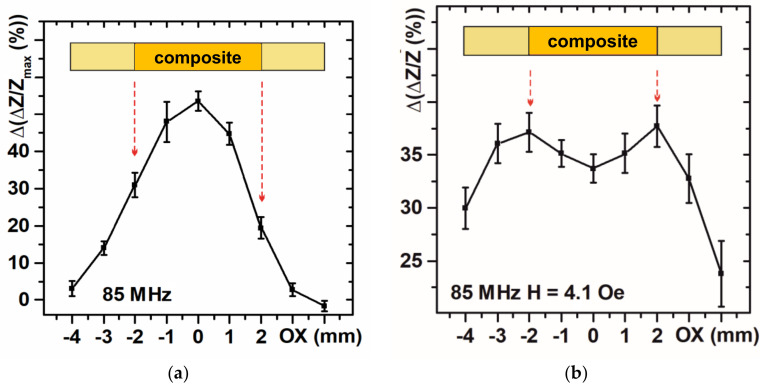
GMI response at the different positions of the magnetic insert: maximum MI ratio (**a**), in the sensitive range of the magnetic field (**b**). Arrows point to the points that correspond to the magnetic insert boundaries.

For the analysis of the maximum MI value, the maximum response of the MI element was observed at the position when the center of the composite was above the center of the MI element: the MI response is almost zero at the magnetic composite position (−4 and 4 mm) (Figure 7a). However, the ΔZ/Z(H) curve maximum is quite wide. For practical reasons, the detection of the maximum is not always the optimum procedure (it requires some iteration measurements for the definition of peak position).

Let us select the sensitive range of the external magnetic field and analyze MI responses for H = 4.1 Oe. At the magnetic composite position (−4 mm, 4 mm) ΔZ/Z(H = 4.1 Oe) ratio value was about 30%. It indicates that, although, magnetic composite is situated far from the center of MI element, even the magnetic stray fields can be easily detected. However, the MI response at the central position of the magnetic insert (0 mm) corresponds to a local minimum and ΔZ/Z(H = 4.1 Oe) was weaker than in the cases for ΔZ/Z_max_ (Figure 7b). The reason can be that the stray magnetic field shifts effective external field point (4.1 Oe) from the sensitive range as the effective field is a superposition of the external field and stray field strength corresponding to each particular space point. Moreover, the perpendicular to the long side of the MI element magnetic stray field component can reduce the working interval of magnetic sensor as well. Therefore, the next step in understanding of the results of the composite detection was made in the direction of the calculation of the geometry of the stray fields of the cylindrical composite.

### 3.3. Magnetic Modeling

**Figure 8:**
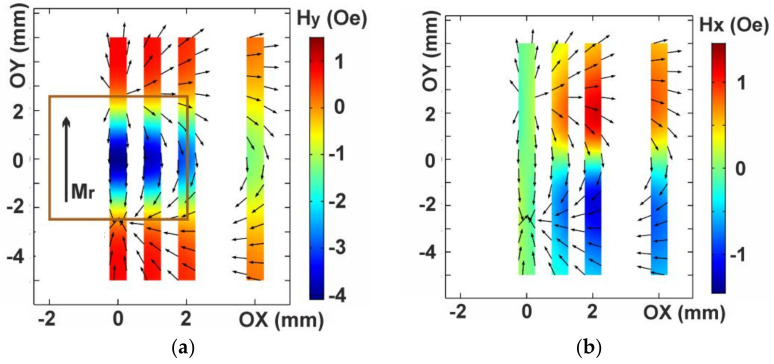
Stray magnetic fields distribution on the surface of the MI multilayered element for different positions of the magnetic composite sample placed at a distance of 1.2 mm from the surface of the MI element. Magnetic composite magnetization is directed along the OY axis: *Hy* stray magnetic field component along the long side of the MI element (**a**); *Hx* stray magnetic field component along the short side of the GMI element (**b**). *Mr*—the magnetization direction of the magnetic composite in XY plane.

According to the Comsol-based modeling, the stray magnetic field distribution is uniform throughout the thickness of thin film element, thus we consider stray magnetic field on the surface of the MI element. As the position of the center of magnetic composite sample approaches the position just above the center of the MI element, the *Hy* stray magnetic field component increases. At the position of the magnetic composite just above the center of the MI element (OX = 0 mm), the stray magnetic field value was as high as about 3 Oe. It was concentrated in the central region of the MI element (±2 mm along OY axis) and it had the opposite sign with respect to the Y coordinate. At the edges of the MI element, stray magnetic fields had the same direction as the OY axis being about 1 Oe. When the magnetic composite position changes away from the center of the MI element, magnetic field concentration region is expanded and the stray magnetic field strength decreases (Figure 8a).

The *Hx* stray magnetic field component is almost zero at the position OX = 0 mm. At the other positions (OX: 1, 2 and 4 mm) stray magnetic field strength was about 1 Oe. It was oriented in opposite directions with respect to the OX axis in symmetric regions around the OY = 0 mm position (Figure 8b). In the presence of the stray magnetic field along the long side of the MI element (along the OY axis) without component along the short side (along the OX axis), the MI curves just shift along the field axis and the maximum MI ratio does not change. In the model magnetic fields distribution at the magnetic composite position just above the MI element *Hx* value is almost zero. However, the MI measurements show the largest decrease in the MI ratio (Figure 6). The reason for this is that the magnetic composite consists of relatively large magnetic particles each of which has an individual magnetic anisotropy axis. Thus, an effective magnetic anisotropy axis for the whole magnetic composite sample is affected by the distribution of the individual axes of the particles. Additionally, as the magnetic composite has a cylindrical form, demagnetization factors contribute to the position of the effective magnetic anisotropy axis as well. Unfortunately, in such circumstances we cannot calculate exact parameters for the direction of the magnetization in the magnetic composite. However, we can suggest some deviation of the cylindrical magnetic composite sample magnetization from the OY axis in the XY plane and describe the result qualitatively.

**Figure 9:**
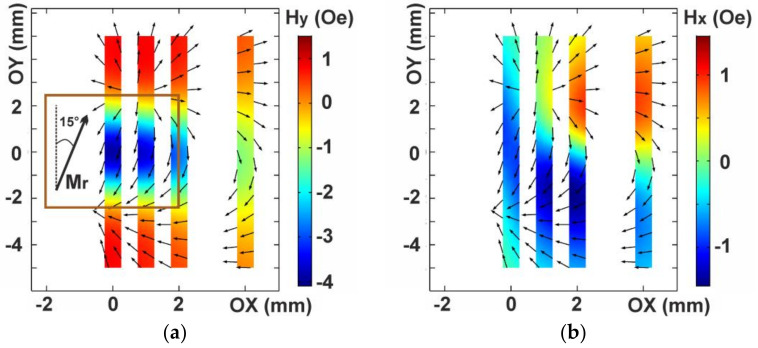
Stray magnetic field distribution on the surface of the GMI element at the different positions of the magnetic insert, the magnetic insert magnetization is directed at an angle 15° related to the OY axis: *Hy* stray magnetic field component along the long side of the GMI element (**a**); *Hx* stray magnetic field component along the short side of the GMI element (**b**). *Mr*—the magnetic insert magnetization direction in the XY plane.

Let us consider that the magnetic composite sample magnetization is directed at an angle of 15° with respect to the OY axis. The *Hy* stray magnetic fields component distribution is almost the same as in the case without magnetization deviation (Figure 9a). However, the *Hx* stray magnetic fields component distribution is significantly different. The *Hx* stray magnetic fields component is about −1 Oe and oriented in the opposite direction with respect to the OX axis throughout the MI element length at the position of the magnetic insert is above the GMI element (OX = 0 mm). At the other positions (OX: 1 mm, 2 mm, 4 mm), magnetic field distribution can be described by selection of two characteristic regions. The magnetic stray field in these regions is oriented in opposite directions along the OX axis (as in the case without magnetization deviation), but these regions have different areas and stray magnetic field strengths. An exception is the position at 4 mm displacement where magnetic field distribution is close to the case without magnetization deviation (Figure 9b).

Since the described theory [21] includes the model with uniformly distributed stray magnetic fields on the surface of the MI element in contrast to our case, it cannot be fully applied there. The results of the magnetic model with the GMI measurements can be interpreted as follows. The maximum MI ratio depends on *Hx* component. If the total field contribution of the *Hx* component is zero, then the maximum MI ratio does not change. For example, at the position OX = 4 mm there are two regions of magnetic field with opposite directions along the OX axis (Figure 9b) and the MI ratio curves just shift due to the *Hy* stray magnetic field component without decrease of the maximum MI ratio. As the magnetic composite approaches the MI element, one of these regions is expanded in area and magnetic field strength in this region increases. This leads to the rise in the total field contribution of the *Hx* component, thus the maximum of the MI ratio decreases. At the position OX = 0 mm the maximum value of the *Hx* component of the stray magnetic field is weaker than in position OX: 1 and 2 mm, however it has one direction throughout the MI element length (Figure 9b) and largest decrease in maximum MI ratio is observed (Figure 6a). If there is no *Hx* component of magnetic field in this position, no change in the maximum of the MI ratio will be observed.

## 4. Discussion

In the previous section, we experimentally demonstrated the validity of proposed approach for detection of the position of magnetic composite by MI magnetic field sensitive element and developed a simple model for understanding of contributions of the stray fields of magnetic particles of the composite at its different positions. 

**Figure 10:**
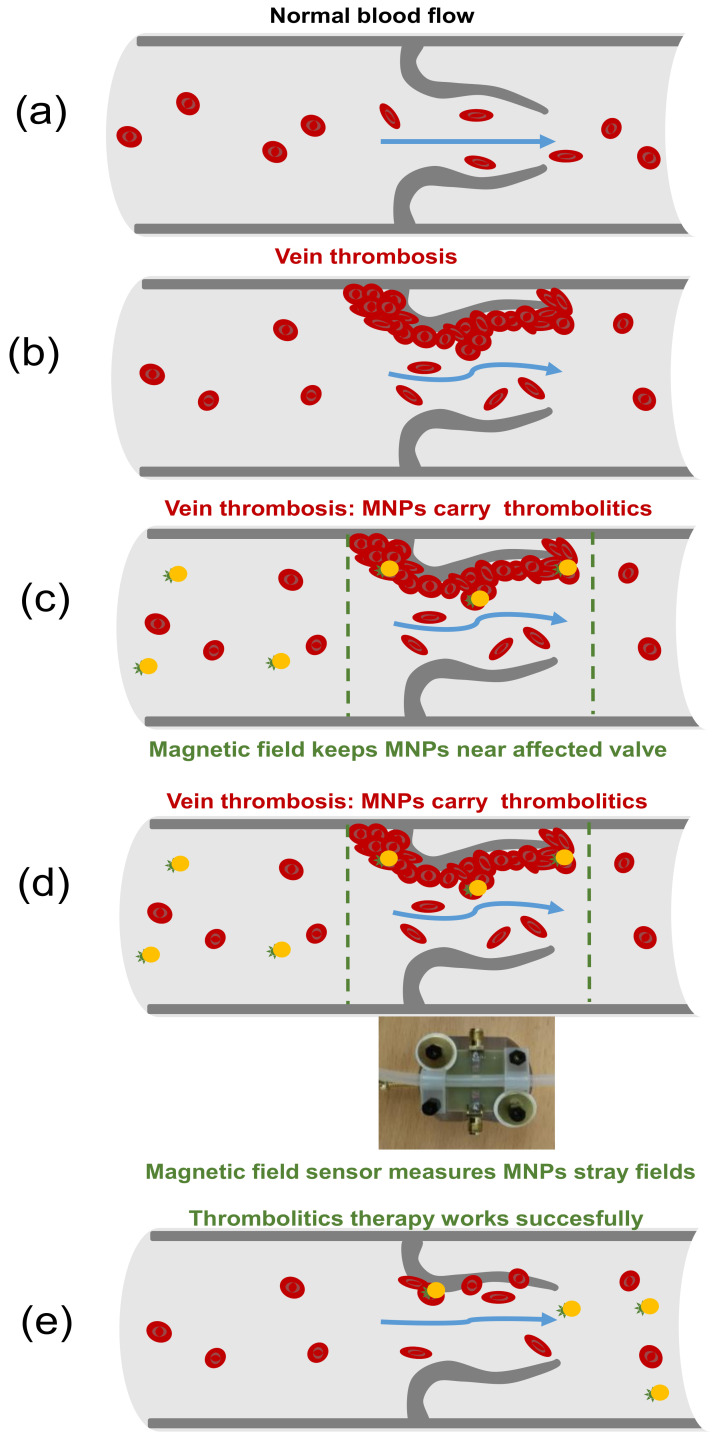
Schematic description of the normal blood flow in a vein (**a**) and blood flow near thrombosis affected vessel (**b**). Intravenous injection of magnetic particles carried on thrombotic drug and directed toward the therapy zone (indicated by vertical dashed lines) by gradient magnetic field (**c**). Magnetic field sensor evaluates the stray fields of magnetic particles accumulating in the therapy zone in a series of displacements measurements, which can be extended by periodic measurements of the response for evaluation of the particles concentration near the blood clot center (**d**). End of the therapy and particles removal (**e**).

Now we would like to discuss some possible directions for future applications of MI sensors for thrombus state evaluation and thrombosis treatment. As it was mentioned in the introduction, there is a visible progress in the therapy with thrombolytic medication, which includes targeted drug delivery to a blood clot using MPs [22]. Figure 10a in schematic way describes the normal blood flow in a vein and Figure 10b part shows the region affected by thrombosis with irregular blood flow. The therapy involving the magnetic nanoparticles allows significantly increased local concentration of the thrombolytic drug immobilized onto their surface by directing them into the thrombosis region by the gradient magnetic field. It is also important to mention that such a procedure results in significant reduction of the time between the intravenous magnetic particles injection and their actuation time. The last means a greater number of the particles not localized by the microphages before their arrival to the actuation point. The main advantage of the whole procedure is a reduction of the side effects. 

However, it is very difficult to evaluate the efficiency of the drug delivery without direct measurement of the number of magnetic particles at the therapy action point. Therefore, we propose to evaluate the amount of the particles there by the measurements of the superposition of their stray fields by magnetic field MI sensor (Figure 10c). Here all results described in the previous section become useful. As soon as the concentration of magnetic particles delivered by applied gradient magnetic field becomes non-zero at the point of therapy the MI sensor can define both the blood clot geometry and particles concentration. To make this possible the scanning procedure must be considered, i.e., one should measure the MI sensor responses at different positions of the device. Exactly as it was demonstrated above in the model experiments with filled composite samples. As it is critically important to determine the local concentration of magnetic particles in the thrombus region during the therapy, after definition of a central point of the blood clot the MI measurements can be periodically made at fixed position corresponding to a center.

Once the therapy has ended and magnetic particles are removed from the blood vessel previously hosting the blood clot, the magnetic field sensor can confirm that the stray field strength (concentration of magnetic particles) becomes very small. On one hand, we should mention the difference between large magnetic particles used for model experiments here and magnetic nanoparticles of the iron oxide usually designed for biomedical applications [5,11,39]. On the other hand, the only difference here would be the smaller value of the stray fields created by nanoparticles. This would require the higher sensitivity of the magnetic field sensor and its operation in shielded environments but such conditions are achievable and become standards of present day applications.

The idea of the addition of the iron oxide MPs to the blood stream is widely discussed in the literature and it is under current investigation for many purposes of theranostics [10,40,41,42]. There are many ways to make magnetic particles useful from magnetic imaging and drug delivery through hyperthermia toward regenerative medicine [39,43,44,45,46]. In this particular study, we just discussed one of possible directions of such applications combining a previously proposed way of the treatment of thrombosis affected vessel [22] with the help of magnetic particles delivering thrombosis drugs. The better way of the treatment control can be achieved by the MI sensor implication. The future will answer the question to what extent this particular approach is realistic and to what extent it can be used for the detection of clusters (agglomerates) of artificially introduced magnetic nanoparticles formed because of specific uptake or magnetic hyperthermia/thermal ablation. It seems to be difficult but possible. The tendency of incorporation of biosensors is increasing [20] and demanding additional efforts for the development of compact analytical devices for therapies and diagnostics.

In addition, it should be mentioned that appearance of nonuniform distribution of the magnetic deposits inside the living system is a quite typical situation. It can be connected with internal biological structures [47] or reflect of the changes of morphology related to the development of particular disease [48,49]. In any case, the technique to define the morphology of such cluster (agglomerate) is highly desired for correct diagnostics.

The idea to use MI effect for the detection of the stray field of magnetic nanoparticles was introduced and proven in 2003 [50] and its validity was confirmed by many studies [51,52,53]. The pico-Tesla MI sensor based on amorphous wire for biomagnetic measurements without magnetic shielding at room temperature was described in 2012 [54]. However, thin film based prototype is the most compatible and requested for existing production techniques, especially for the case of magnetic label or clusters detection [17]. Despite the fact that thin film based MI sensitive elements were intensively studied in the last 10 years and very high sensitivities of the order of 300%/Oe were obtained [55], promising approaches are under development. For example, a new type of MI multilayered structures with a high MI sensitivity has been proposed recently [56]. The multilayer structure consists of a highly conductive central layer and two outer ferromagnetic layers located below and above the conductive layer. The upper layer is a periodic structure, of N-multilayered elements and N + 1-regions, in which multilayered elements are absent (the upper layer is profiled). It is shown that for a profiled structure with a decrease in the deviation angle of the effective magnetic anisotropy axis from the transverse direction, the magnetic permeability of the upper layer increases, which leads to an increase in the skin effect and an increase in the MI effect. The progress in the development of the MNPs with lower size distribution and improved properties is continuous [18,44,45]. Both increase of the magnetic field detector sensitivity and enhanced quality of magnetic carriers contribute to the hope that proposed model experiments become a reality soon.

## 5. Conclusions

Magnetic multilayered [FeNi (100 nm)/Cu (3 nm)]_5_/Cu (500 nm)/[Cu (3 nm)/[FeNi (100 nm)]_5_ MI sensitive element was prepared by magnetron sputtering deposition in the external magnetic field in order to create well defined induced magnetic anisotropy. Magnetic properties, magnetization process peculiarities and magnetoimpedance effect were carefully analyzed. For model experiments non-ferromagnetic polymer tube was used to design the sample mimicking thrombus in a blood vessel. Cylinder sample was made from epoxy resin with a magnetic insert placed in the center part-filled composite type material containing 30% weight concentration of commercial magnetic iron oxide microparticles. The detection of the position of the magnetic composite mimicking thrombus in the blood vessel was carried out by the measurements of the stray magnetic fields of iron oxide microparticles using magnetoimpedance sensitive element. Changes of the MI ratio in the presence of composite can be characterized by the shift and the decrease of the maximum value of the MI. The magnetic field sensitivity of the longitudinal MI effect strongly depends on the value of the external field. The longitudinal component of the field can shift working point from the sensitive range of the field and transverse component can decrease sensitivity. We were able to detect the position of the magnetic composite sample mimicking thrombus in blood vessels. Comsol modeling of stray fields was successfully used for the analysis of the obtained experimental results. Although the model is rather simple, it provides good understanding of the origin the MI sensitivity in the proposed configuration. We describe a possible use of MI sensors for biomedical applications in the field of thrombus state evaluation and therapy.

## Data Availability

Data available from the corresponding author upon reasonable request.
